# Effect of Growth Temperature on the Characteristics of CsPbI_3_-Quantum Dots Doped Perovskite Film

**DOI:** 10.3390/molecules26154439

**Published:** 2021-07-23

**Authors:** Shui-Yang Lien, Yu-Hao Chen, Wen-Ray Chen, Chuan-Hsi Liu, Chien-Jung Huang

**Affiliations:** 1 School of Opto-Electronic and Communication Engineering, Xiamen University of Technology, Xiamen 361024, China; sylien@xmut.edu.cn; 2 Department of Materials Science and Engineering, Da-Yeh University, Dacun, Changhua 51591, Taiwan; 3 Fujian Key Laboratory of Optoelectronic Technology and Devices, Xiamen University of Technology, Xiamen 361024, China; 4 Department of Applied Physics, National University of Kaohsiung, Kaohsiung University Rd., Kaohsiung 81148, Taiwan; a0976840286@gmail.com; 5 Department of Electronic Engineering, National Formosa University, Wenhua Rd., Yunlin County 632301, Taiwan; chenwr@nfu.edu.tw; 6 Department of Mechatronic Engineering, National Taiwan Normal University, Heping East Rd., Taipei 10610, Taiwan; liuch@ntnu.edu.tw

**Keywords:** perovskite, solar cell, quantum dots, hot injection, mobility, thermal stability

## Abstract

In this study, adding CsPbI_3_ quantum dots to organic perovskite methylamine lead triiodide (CH_3_NH_3_PbI_3_) to form a doped perovskite film filmed by different temperatures was found to effectively reduce the formation of unsaturated metal Pb. Doping a small amount of CsPbI_3_ quantum dots could enhance thermal stability and improve surface defects. The electron mobility of the doped film was 2.5 times higher than the pristine film. This was a major breakthrough for inorganic quantum dot doped organic perovskite thin films.

## 1. Introduction

Organic perovskite CH_3_NH_3_PbI_3_ (MAPbI_3_) is considered to be the most potential light-absorbing material for perovskite solar cells (PSCs) due to its high optical absorption characteristics and long diffusion length [1]. Compared with silicon solar cells, and although they dominate the solar industry with efficiencies of over 20%, silicon solar cells remain relatively expensive to manufacture [2]. In the industry, in order to ensure large-scale production and meet future energy consumption needs, there is an urgent need to significantly reduce manufacturing costs. In recent years, perovskite solar cells (PSCs) have received widespread attention based on very low material costs. According to previous reports, the conversion efficiency of organic perovskite solar cells has rapidly increased from 9.2% to 20.5% [3,4], and the mobility of perovskite samples is calculated to be 60–75 cm^2^ V^−1^ s^−1^ [5]. However, organic perovskite MAPbI_3_ still has many problems that need to be overcome. For example, it is easily degraded for organic perovskite in air and the hygroscopicity of methylammonium (MA) cations will trap moisture in the air, which will increase the crystal size and cause pollution [6,7]. Therefore, improving the organic perovskite MAPbI_3_ has become a concern in recent years. Amalie Dualeh et al. used control of the film formation temperature to improve the photoelectric conversion efficiency (PCE) of MAPbI_3_ [8]; Xiao Bing et al. used inorganic PbCl_2_ to increase the carrier mobility of perovskite solar cells [9]; LC Chen et al. used doped FAPbI_3_ quantum dots (QDs) to enhance the photoelectric conversion efficiency of MAPbI_3_ [10]. It can be found that passivation treatment and doping with inorganic materials have become an important basis for improving organic perovskite MAPbI_3_. Based on the above, doping inorganic quantum dots (CsPbI_3_) into MAPbI_3_ is still poorly studied. Therefore, in this article, a detailed investigation of improvements in the light-absorption capacity and carrier mobility of MAPbI_3_ by doping with inorganic quantum dots CsPbI_3_ and changing the filming temperature is presented.

## 2. Results

**Figure 1:**
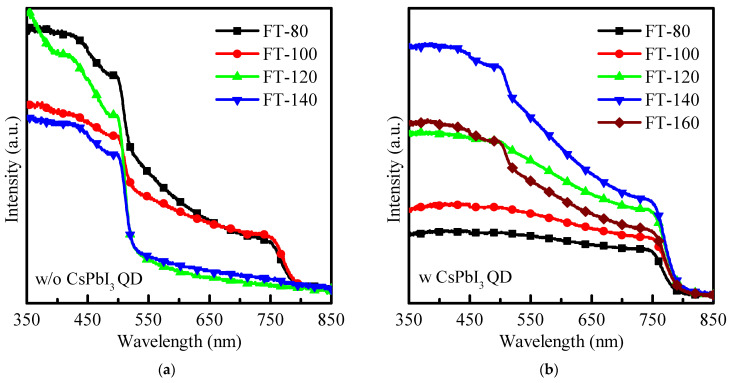
(**a**) Absorption spectrum of pristine MAPbI_3_. (**b**) Absorption spectrum of CsPbI_3_ -QD doped MAPbI_3_ under different filming temperatures (FTs) from 80 to 160 °C.

As shown in Figure 1a, when the filming temperature is 80–100 °C, pristine MAPbI_3_ can still show a typical perovskite absorption spectrum; however, when the filming temperature is further heated to 120–140 °C, the pristine MAPbI_3_ shows a significant decrease in the absorption spectrum, and the decomposed to PbI_2_ phase dominated. The decomposition of MAPbI_3_ can change from dark brown to yellow, similar to previous reports [11,12]. Figure 1b shows the absorption spectrum for CsPbI_3_-QD doped perovskite thin films. It can be found that when the filming temperature exceeds 120 °C, the typical perovskite absorption peak can still be observed at 750 nm. This is due to the addition of CsPbI_3_-QDs, which stabilize the structure of the perovskite film surface and make MAPbI_3_ difficult to degrade. In addition, after increasing the filming temperature, the absorption area increases significantly in the entire spectral range (350–850 nm), and the long-wavelength absorption (750 nm) is significantly improved. This is because the energy gap of CsPbI_3_ QD is wider and a small strain occurs at the QDs–MAPbI_3_ interface [12,13,14]. Therefore, adding CsPbI_3_ QDs can not only stabilize the MAPbI_3_ film at a higher filming temperature, but also improve the absorption of the film at long wavelengths, and further enhance the absorption capacity of CsPbI_3_-QD doped perovskite thin films in the active layer of perovskite solar cells.

**Figure 2:**
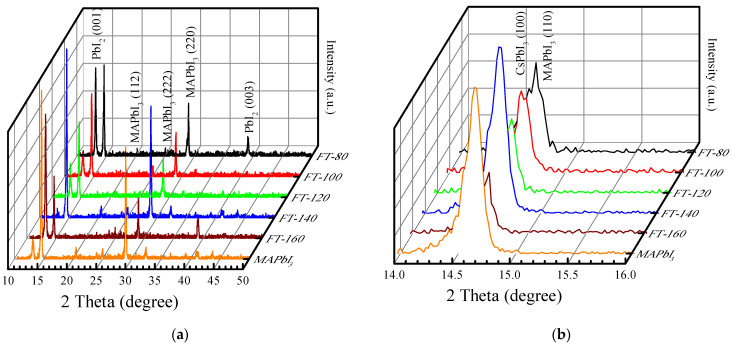
(**a**) XRD patterns of CsPbI_3_-QD doped perovskite thin films under 80–160 °C. (**b**) XRD patterns under the scale of 14°~16°.

Figure 2a demonstrates the X-ray diffraction (XRD) pattern of CsPbI_3_-QD doped perovskite thin films when the filming temperature is 80–160 °C. Based on the spectra of conventional MAPbI_3_ films [14], the peak position for MAPbI_3_ in CsPbI_3_-QD doped perovskite thin films under different filming temperatures appeared at 14° and 28° and all the films demonstrated strongest intensity along (110). There is an additional new peak at 12.7°, which is attributed to PbI_2_. The intensity of the PbI_2_ peak of the control sample (pristine MAPbI_3_) is much greater than that of BT-140, and there is almost no PbI_2_ peak in FT-140. This is due to the better thermal stability that effectively inhibits the formation of PbI_2_ and the doping of CsPbI_3_ QDs avoids the degradation of MAPbI_3_ which is due to the decrease in hydrogen bonds in MAPbI_3_ and the increase in the octahedral tilt due to the Cs-ion exchange process [15]. When the filming temperature is increased to 160 °C, the peak intensity of PbI_2_ (001) is much stronger than the perovskite peak. Generally, the change in the filming temperature can be used to remove impurities or organic substances from the surface of the film to optimize it. When the filming temperature is lower than 140 °C, excess ligands (oleylamine, oleic acid) or PbI_2_ is removed, but when the filming temperature is 160 °C, MAPbI_3_ degrades, resulting in a large amount of PbI_2_ that will damage the structure of the doped thin film. Figure 2b shows the details of the preferred peaks of the QD doped film. According to previous research, it is found that when the filming temperature is up to 140 °C, the ratio of the peak area CsPbI_3_/MAPbI_3_ is close to 1 and the perovskite crystallinity is optimal [12].

**Figure 3:**
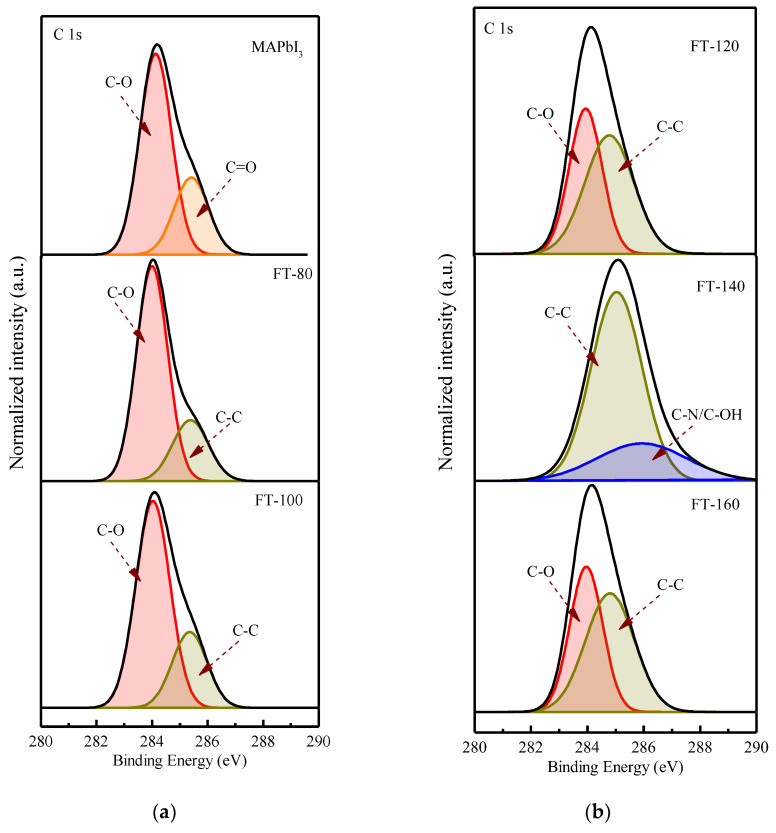
XPS core-level spectra of C 1s (**a**) Pristine MAPbI_3_ and CsPbI_3_-QD doped MAPbI_3_ under different filming temperatures (FTs) from 80 to 100 °C. (**b**) CsPbI_3_-QD doped MAPbI_3_ under different filming temperatures (FTs) from 120 to 160 °C.

In order to explain the charge recombination effect introduced by CsPbI_3_-QDs, the XPS spectrum of the film was measured and it was understood that changing the film formation temperatures may affect the surface stability of the MAPbI_3_ film. Figure 3 shows the core-level spectra of CsPbI_3_-QD doped perovskite thin films at different filming temperatures. The deconvolution characteristic of the carbon peak shows the binding state of carbon material and atmospheric oxygen. The peak at 283.97 eV corresponds to C-O and the peak at 285.4 eV corresponds to C=O [16]; the carbon configuration combined with oxygen can be found in the spectra of the control group (pristine MAPbI_3_), which is due to the moisture absorption of the MAPbI_3_ film when it is exposed to air and the perovskite thin films surface will be oxidized; therefore, it will lead to the appearance of a C-O peak and C=O peak. After adding CsPbI_3_-QDs, the C=O peak disappeared and was converted to a C-C peak; even after the filming temperature was increased to 140 °C, the C-O peak disappeared. This could be due to the higher temperature which will eliminate the weakly bound organic components.

**Figure 4:**
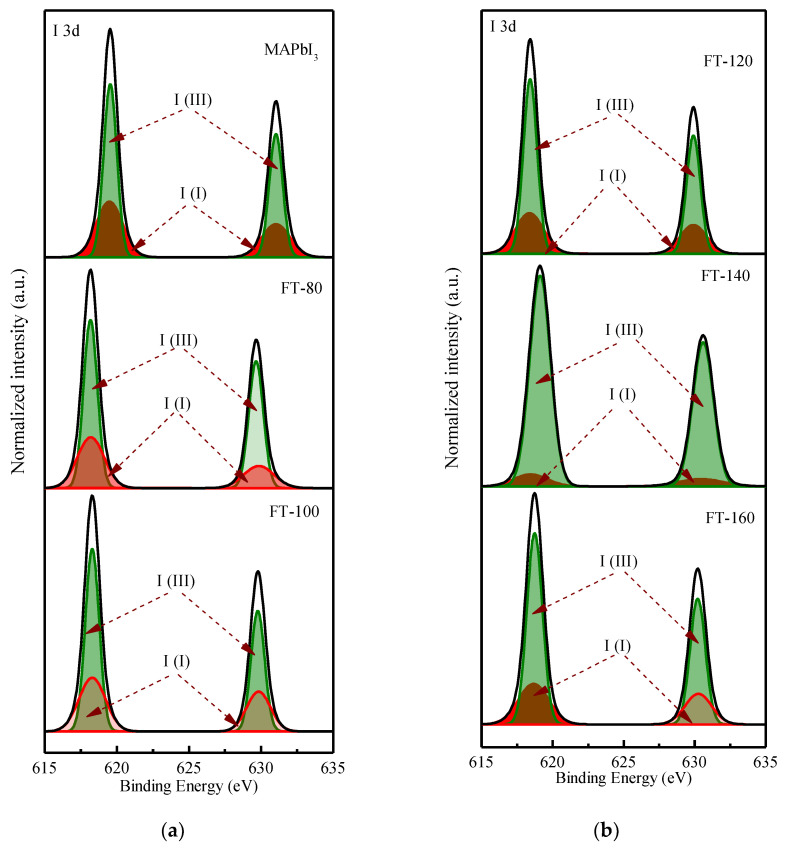
XPS core-level spectra of I 3d (**a**) Pristine MAPbI_3_ and CsPbI_3_ -QD doped MAPbI_3_ under different filming temperatures (FTs) from 80 to 100 °C. (**b**) CsPbI_3_ -QD doped MAPbI_3_ under different filming temperatures (FTs) from 120 to 160 °C.

**Figure 5:**
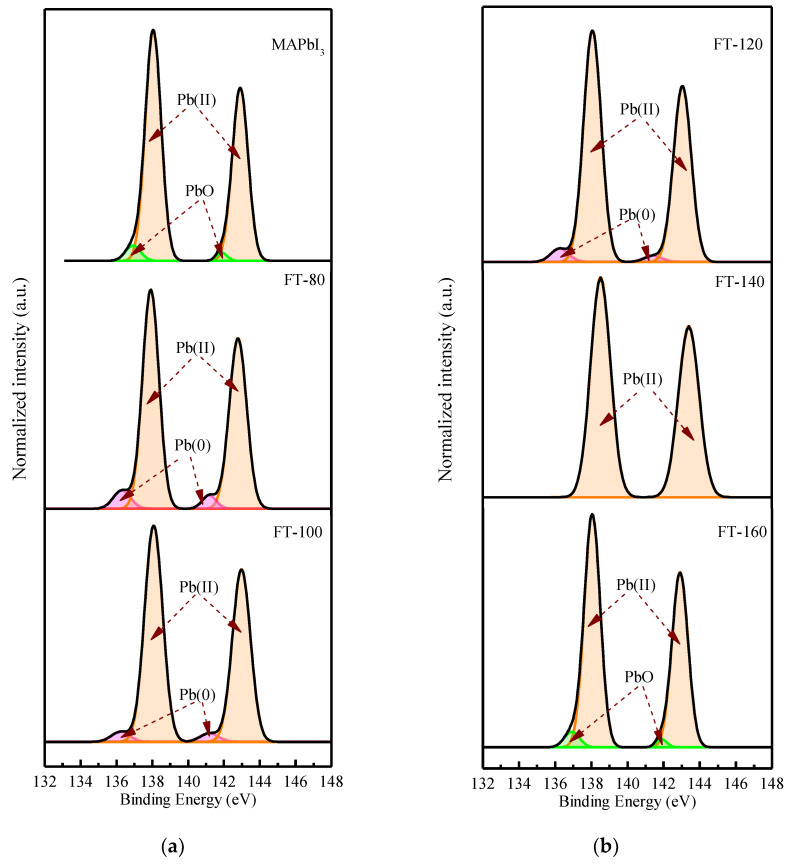
XPS core-level spectra of Pb 4f (**a**) Pristine MAPbI_3_ and CsPbI_3_ -QD doped MAPbI_3_ under different filming temperatures (FTs) from 80 to 100 °C. (**b**) CsPbI_3_ -QD doped MAPbI_3_ under different filming temperatures (FTs) from 120 to 160 °C.

Figure 4 shows the deconvoluted XPS spectrum of the I 3d doublet. The values of 619.5 and 631 eV correspond to the I_3_^−^ charge state, while 619.37 and 630.87 eV correspond to the I^2+^ charge state. Figure 5 shows the deconvoluted XPS spectrum of the Pb 4f doublet. The values of 136.23 and 141.18 eV correspond to metallic lead (Pb), while 138.07 and 142.97 eV correspond to Pb (II) in perovskite. From the Pb XPS spectra, it can be found that after adding quantum dots and increasing the film forming temperature, the percentage of Pb (II) species is relatively higher than that of metal Pb, even if the temperature is increased to 160 °C. This shows that the iodine atom interacts with the lead atom and forms a donor–acceptor complex. This is because the low electronegativity Pb atom provides the excess unpaired electrons to the high electronegativity I(I), and in the process of electron transfer, the Pb atom is oxidized to Pb^2+^ and provides two electrons to reduce the iodine atom to 2I^−^, and is further reduced to triiodide(I_3_^−^). It can be clearly understood by the following equation:

$$\mathrm{Pb}\to {\mathrm{Pb}}^{2+}+2e^{-}$$

$$I+2e^{-}\to 2I^{-}$$

$$I_{2}+I^{-}\to I_{3}^{-}$$

However, when the filming temperature is increased to 140 °C, the peak of metallic lead disappears. Recent studies have shown that the peak of metallic lead is derived from unsaturated lead, and the presence of unsaturated lead atoms is related to the lack of iodide [17], and the metal lead is compounded as recombination point, leading to poor performance. Due to its thermal stability, Cs atoms replace some MA, resulting in the loss of molecular groups and fewer iodine atoms at the A site of the perovskite, and unsaturated Pb is effectively suppressed.

**Figure 6:**
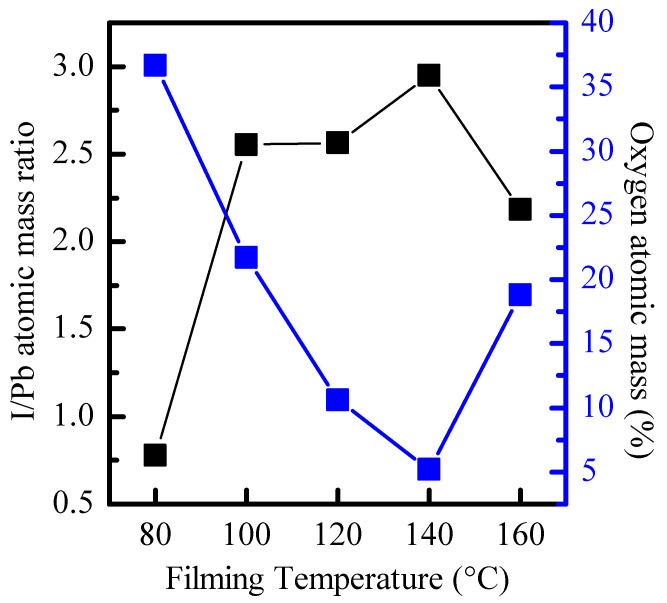
Quantified XPS results highlighting atomic mass ratio for I/Pb and oxygen atomic mass percentage for different filming temperatures.

Figure 6 shows the relationship between the I/Pb atomic mass ratio calculated from the integral area of Pb 4f and I 3d and the total atomic mass percentage of O 1s and the filming temperature. Research has pointed out that the thickness of the film is related to the combination of surface oxygen [18]; however, the thickness of the film is 295 nm at different filming temperatures. Therefore, it can be further inferred that the total atomic concentration of the I 3d peak gradually increases relative to the total concentration of the Pb 4f peak, which is related to the reduction in surface oxides. Therefore, it can be seen that when the filming temperature is 140 °C (I/Pb ratio is closest to 3), the CsPbI_3_-QD doped perovskite thin films surface can be effectively stabilized and prevented from oxidation.

It can be found from Table 1 that after the filming temperature is increased, the mobility is significantly increased. This is attributed to the addition of CsPbI_3_ QDs, which effectively prevents the formation of metallic lead and reduces the chance of electron-hole recombination.

## 3. Materials and Methods

### 3.1. Materials

All materials contain cesium carbonate (Cs_2_CO_3_, 99.9%), lead(II) iodide (PbI_2_, 99.9985%), oleic acid (C_18_H_34_O_2_, analytical reagent 90%), oleyl amine (C_18_H_35_NH_2_, 90%), 1-octadecene (ODE, technical grade 90%), toluene (anhydrous, 99.8%), hexane (analytical reagent, 97%), methyl acetate (MeOAc, anhydrous 99.5%), methylammonium iodide (CH_3_NH_3_I, 99%), dimethyl sulfoxide ((CH_3_)_2_SO, 99%) and gamma-butyrolactone (C_4_H_6_O_2_, 99.9%), as shown in Table 1. All the chemicals in this work were used without further treatment.

### 3.2. Solution Preparation and Synthesis for Cs-Oleate Precursor, CsPbI_3_ QDs and CH_3_NH_3_PbI_3_

The experimental method is the modified hot-injection method previously reported [12]. All experiments were performed in a glove box filled with nitrogen, H_2_O < 1 ppm and O_2_ < 1 ppm.

### 3.3. Synthesis of Cs-Oleate

Cs_2_CO_3_ (0.1 g), OA (0.5 mL) and ODE (10 mL) were loaded into a 50 mL sample bottle and stirred for 1 h at 120 °C. We used heating and air extraction to remove moisture and internal air. Then, the solution was heated at 150 °C until the solution was clear. Finally, the Cs-oleate was stored at 100 °C to avoid precipitation.

### 3.4. Synthesis of CsPbI_3_ QDs

Both ODE (10 mL) and PbI_2_ (0.173 g) were added into a 50 mL sample bottle and were dried at 120 °C for 1 h. Then, 1 mL of OA and 1 mL of OAM (preheated at 70 °C) were poured. The solution was degassed until the PbI_2_ completely dissolved and the solution became clear. The solution was then heated to 185 °C. The Cs-oleate (0.0625 M, 1.6 mL) precursor was swiftly injected into the solution. After 5 s, the reaction solution was cooled by immediately immersing the sample bottle into an ice bath.

### 3.5. Purification of CsPbI_3_ QD

The prepared CsPbI_3_ QDs were separated by adding MeOAc (volume ratio of crude solution/MeOAc is 1:3), and then they were centrifuged at 8000 rpm for 5 min. After that, the supernatant was discarded, and the precipitation that contained the QDs was dissolved in 3 mL of hexane. Then, the CsPbI_3_ QDs were precipitated again by adding MeOAc (volume ratio of crude solution/MeOAc is 1:1) and centrifuging at 8000 rpm for 2 min. Finally, the QDs were dispersed in 3 mL of hexane and centrifuged at 4000 rpm for 5 min to remove excess PbI_2_ and precursor.

### 3.6. Synthesis of CH_3_NH_3_I

We added CH_3_NH_3_I (198.75 mg) and PbI_2_ (576.25 mg) into the 50 mL sample bottle, and then added C_2_H_6_OS (0.5 mL) and C_6_H_6_O_2_ (0.5 mL) into the sample bottle in the glove box and stirred at 300 rpm for 24 h.

### 3.7. Fabrication of Thin Films

CH_3_NH_3_I (50 μL) and CsPbI_3_ (1 mg) were mixed and spin-coated on the glass substrate in the glove box and then filmed by different filming temperatures from 80–160 °C.

### 3.8. Characteristic Measurements

The absorption spectra of the thin film were measured by ultraviolet/visible (UV/vis) absorption spectroscopy (HITACHI, U-3900, Hitachi High-Technologies Corporation Tokyo, Japan). X-ray diffraction (XRD) data of films were recorded by the Bruker D8 Discover (Bruker AXS Gmbh, Karlsruhe, Germany) X-ray diffractometer with Grazing Incidence X-ray Diffraction (GIXRD) and X-ray photoelectron spectroscopy (XPS) data of films were recorded by a PHI 5000 (ULVAC-PHI, Kanagawa Prefecture, Japan) VersaProbe/Scanning ESCA Microprobe.

## 4. Conclusions

We successfully manufactured CsPbI_3_-QD doped perovskite thin films and clearly analyzed the surface of this film through an XPS core-level configuration. By increasing the temperature of film formation, the light-absorption capacity can be effectively improved and the precursors and organics can be reduced. The doping of a small amount of CsPbI_3_ QDs can reveal better thermal stability to improve the surface trap state. Therefore, this kind of QD doped perovskite thin film will become an important key to improve the efficiency of perovskite solar cells in the future.

## Figures and Tables

**Table 1:** The mobility, resistivity and carrier concentration of the control group and different filming temperatures.

	Mobility (cm^2^/V_s_)	Resistivity (cm^2^/C)	Carrier Concentration (cm^−2^)
pristine MAPbI3	$1.95\times 10^{3}$	$8.86\times 10^{8}$	$3.61\times 10^{6}$
FT-80	$1.97\times 10^{3}$	$8.84\times 10^{8}$	$3.65\times 10^{6}$
FT-100	$2.45\times 10^{3}$	$5.35\times 10^{8}$	$3.48\times 10^{6}$
FT-120	$3.46\times 10^{3}$	$3.78\times 10^{8}$	$3.27\times 10^{6}$
FT-140	$4.91\times 10^{3}$	$2.67\times 10^{8}$	$6.97\times 10^{6}$
FT-160	$3.34\times 10^{3}$	$2.75\times 10^{8}$	$4.62\times 10^{6}$

## Data Availability

The data presented in this study are available on request from the corresponding author.

## Sample Availability

Not available.
